# Improved Mechanical and Physical Properties of Epoxy Acrylate Oligomers by Chemical Modification for the Effective Encapsulation of the Triple-Cation Perovskite Solar Cells

**DOI:** 10.1021/acsomega.5c00860

**Published:** 2025-05-07

**Authors:** Bahar Tosun Ercan, Adem Mutlu, Sirin Siyahjani Gultekin, Burak Gultekin, Haluk Dincalp, Ceylan Zafer

**Affiliations:** † 37509 Ege University, Solar Energy Institute, 35100 Izmir, Türkiye; ‡ Kubilay Paint Industry, 35800 Izmir, Türkiye; § Manisa Celal Bayar University, Department of Chemistry, Faculty of Arts and Science, Yunus Emre, 45140 Manisa, Türkiye

## Abstract

This study modifies epoxy acrylate to enhance its mechanical, thermal, and barrier properties, such as hardness, flexibility, gloss, adhesion, and water/oxygen resistance. Adipic acid (AdAc) and 3-aminotriethoxysilane (ATES) were incorporated into the epoxy acrylate structure, and the resulting oligomers were characterized by Fourier transform infrared (FTIR) and nuclear magnetic resonance (NMR) spectroscopy. Thermal analysis showed that AdAc-modified epoxy acrylate oligomer-3 (AdAc-MO3) (1.70% AdAc) had a lower glass transition temperature (*T*_g_) of 48.2 °C, improving flexibility, while ATES-modified epoxy acrylate oligomer-5 (ATES-MO5) (1.70% ATES) exhibited a higher *T*_g_ of 56 °C, enhancing thermal stability. AdAc-MO3 achieved excellent mechanical and barrier performance, with a water vapor transmission rate (WVTR) of 7.46 g/m^2^day and an oxygen transmission rate (OTR) of 5.10 g/m^2^day. Mechanical tests confirmed that AdAc-MO3 balanced hardness and flexibility, passing adhesion and conical mandrel tests without deformation. The encapsulants were tested on perovskite solar cells (PSCs) with an FTO/Li-TiO_2_/perovskite/Spiro-OMeTAD/Au configuration. After 48 h of stability testing under 60% humidity, 25 °C, and a light intensity of 100 mW/cm^2^, AdAc-MO3 retained 26.8% of its initial power conversion efficiency (PCE), compared to 20.5% for the control device. ATES-MO3 retained 56.1% of its initial PCE, outperforming both the control and AdAc-MO3, but its higher cross-linking density reduced adhesion and flexibility, limiting its use in certain encapsulation applications. Visible light curing further improved stability, reducing efficiency loss to 8% compared to 16% with UV curing. These results demonstrate that AdAc-MO3 is a promising encapsulant for PSCs, combining enhanced properties and stability under realistic conditions.

## Graphical abstract

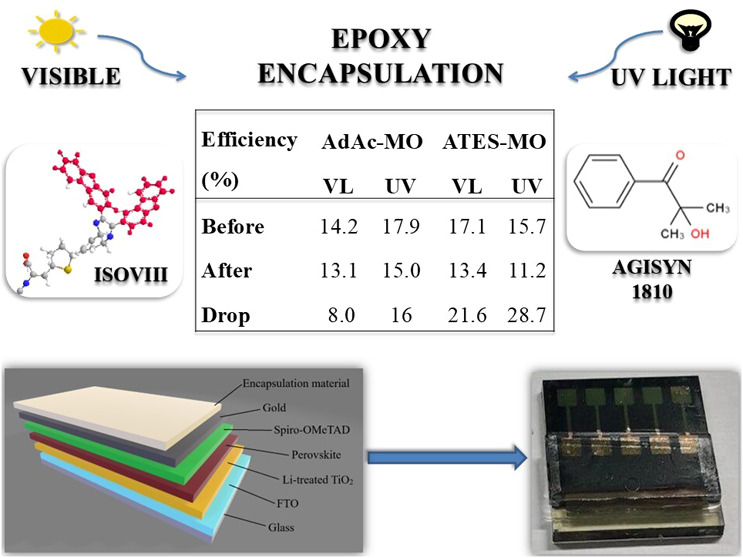

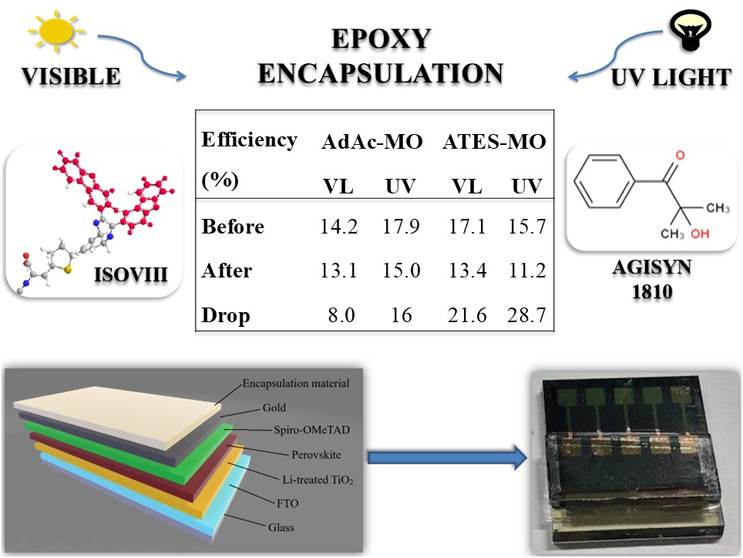

## 1 Introduction

Perovskite solar cells (PSCs) have emerged as a promising technology in the solar cell market due to their low cost, easy processing, and high efficiency (26.7%).^1–4^ Despite efforts to enhance the stability of perovskite materials against moisture, oxygen, irradiation, and temperature, achieving long-term stability for PSC devices in real field operations remains a challenge. This lack of stability compared with silicon solar cells, which dominate the photovoltaic panel market, hinders the commercialization of PSCs.

The encapsulation process plays a crucial role in improving device stability by protecting PSCs from environmental conditions. Encapsulation coatings act as a barrier layer, preventing the diffusion of oxygen and moisture into the active layers of PSCs. Various encapsulation materials have been used for PSCs, including polyvinyl butyral (PVB), ethyl vinyl acetate (EVA), and epoxy derivatives. These materials are preferred for their high chemical and mechanical resistance, thermal stability, low permeability to oxygen and moisture, as well as their cost-effectiveness and ease of processing.^5–11^ Additionally, UV-curable epoxy encapsulation systems have gained popularity due to their rapid curing, low energy consumption, and absence of volatile components harmful to the environment^12^

Previous studies have investigated the effects of epoxy resin as an edge adhesive on the UV-light degradation of cell efficiencies.^13^ Matteocci et al. demonstrated the stability of devices using an encapsulant system comprising UV epoxy edge adhesive, white light-cured adhesive, and a glass surface, showing a maintained stability of 80% after a 100 h stability test at 40–50 °C.^14^ Dong et al. compared three types of epoxy resins (thermal curing epoxy, 2-component epoxy, and UV-curable epoxy) as encapsulants and found that the UV-curable epoxy coating achieved the longest device lifetime.^15^ Li et al. suggested that using Surlyn (Dow Chemicals) and UV-curable epoxy encapsulation materials could enhance stability, maintaining 92% of the initial performance of the device after three months.^16^ Many reports in the literature have discussed extending the lifetimes of PSCs through the use of hydrophobic structures such as UV-curable epoxy, alkyl alkoxysilane, thiols, and oleic acid.^17^ Uddin et al. studied the effects of epoxy, Surlyn, EVA, and poly­(vinyl chloride) on device lifetime and power conversion efficiency (PCE).^18^ Shi et al. proposed the use of polyisobutylene (PIB) as an encapsulant, which maintained the initial device efficiency for up to 540 h.^19^ Additionally, Ramasamy et al. demonstrated that epoxy outperformed Surlyn as an encapsulant, improving device stability beyond 70 days.^6^ Improving the mechanical–chemical strength and water–oxygen transmission rate performance of UV-curable encapsulant epoxy coatings in solar cell encapsulation technology is crucial. Numerous studies have focused on the chemical modification of epoxy resins and oligomers at different ratios. Bisphenol A-based acrylated epoxy oligomers have been synthesized and utilized to improve adhesion.^20–25^ Epoxy acrylate oligomers have been modified with varying amounts of 3-isocyanatopropyl trimethoxysilane (IPTMS).^26^ In other research, epoxy acrylate oligomers were modified with adipic acid (AdAc) and methacrylic acid to enhance the mechanical properties of the resins.^27^

A modified epoxy acrylate oligomer with AdAc and 3-aminotriethoxysilane (ATES) was synthesized by using acrylic acid (AcAc). AcAc is a more commercially available and cost-effective chemical compared with methacrylic acid. Additionally, methacrylic acid provides flexibility to the structure by reducing cross-linking, which, in turn, reduces thermal and chemical resistance. Here, we used AdAc to ensure adhesion to glass; however, when combined with methacrylic acid, flexibility increases further, compromising the mechanical strength properties of the encapsulation material.^28^ Moreover, methacrylic acid reacts more slowly than AcAc, resulting in a longer reaction time.^29^ This prolongs the polymerization conversion and the curing phase of the material. This modified oligomer aimed to prevent the easy breaking of UV-curable epoxy resins and improve their adhesion to glass. The influence of the amounts of AdAc and ATES on the thermal and mechanical properties of the coating was investigated. Additionally, to address UV light degradation caused by the UV-curable encapsulant in PSC structures, a transparent coating based on the modified epoxy acrylate oligomer was cured under visible light using a new photoinitiator (3E)-6,60-bis­(9-ethyl-9H-carbazole-3-yl)-1-[(2R)-2-ethylhexyl]-10-[(2S)-2-ethylhexyl]-3,30-biindole-2,20 (1H,10 H)-dione (ISOVIII) system that absorbs light in the visible spectrum. Electrical parameter analysis of PSCs encapsulated with the synthesized oligomers, cured using sunlight and ultraviolet (UV) light showed us that visible light curing further improved device stability, reducing the efficiency drop from 16% to 8% compared to UV cured devices.

## 2 Materials and Methods

### 2.1 Materials

The bisphenol A/F-based epoxy resin (Er) with an epoxy content of 5260–5420 mmol/kg was supplied by Paksoy Kimya, Turkey. AcAc and AdAc were purchased from Veskim, while ATES was obtained from EvonikDegussa Turkey and used without further treatment. Triethylamine (TEA) and N-N-dimethylbenzilamine (DMBA) were acquired from Fluka. Dipropylene glycol diacrylate (DPGDA, AgiSyn 2833) was obtained, and 2-Hydroxy-2-methyl-1-phenyl-propane-1-one (Agisyn 1810) was purchased from AGI Corporation. Silicon-free defoamer (solution of polyolefin) was supplied by BYK-Chemie GmbH. (3E)-6,60-bis­(9-ethyl-9H-carbazole-3-yl)-1-[(2R)-2-ethylhexyl]-10-[(2S)-2-ethylhexyl]-3,30-biindole-2,20 (1H,10 H)-dione (ISOVIII) is synthesized by our previous study.^30^ Glass slides measuring 2.5 × 2.5 cm^2^ and aluminum sheets were utilized as substrates for all coating applications. Fluorine-doped tin oxide (FTO) glass slides with a sheet resistance of 14 Ω sq^–1^ were procured from OPVTech. Lead (II) iodide (PbI_2_, 99.99%) was purchased from Tokyo Chemical Industry (TCI), while lead (II) bromide (PbBr_2_) (99.999%), methylammonium bromide (MABr, > 99.5%), and formamidinium iodide (FAI, > 99.5%) were obtained from Lumtec. Bis­(trifluoromethane) sulfonimide lithium salt (Li-TFSI, 99.0%) and N,N-dimethylformamide (DMF, anhydrous >99.5%) were purchased from Acros. Dimethyl sulfoxide (DMSO, > 99.7%) was bought from Merck. Titanium isopropoxide (Ti­[OCH­(CH3)_2_]_4_, 97%), hydrochloric acid (HCl, 37%), 2-propanol (for HPLC, 99.9%), 4-*tert*-butylpyridine (TBP, 98.0%), acetonitrile (99.8%), cesium iodide (CsI, 99.999%), and chlorobenzene (anhydrous 99.8%) were acquired from Sigma-Aldrich. Spiro-OMeTAD was purchased from Borun New Material Technology.

### 2.2 Characterization Techniques

Fourier transform infrared spectroscopy **(**FTIR) spectrum was recorded using a PerkinElmer 8303 FTIR spectrometer. The acid value of the samples was determined by dissolving them in a mixture of toluene and ethanol in equal volumes and titrating the resulting solution with a 0.1 mol/L KOH solution at room temperature, using phenolphthalein as an end point indicator.^31^ To assess the coating properties of the cross-linked films, the coating formulations were applied to glass and aluminum substrates using a 30 μm four sided (30, 60, 90, 120 μm) high viscosity applicator and cured in a bench-type UV processor (EMA, Turkey) equipped with medium-pressure mercury UV lamps with a wavelength maximum (λ_max_) of 365 nm and an intensity of 120 W/cm. The coating properties were evaluated according to the corresponding standard test methods. Viscosity of resins was measured by using DIN Cup 6 (hole radius 6 mm) flow time with DIN 53211 standard and converted cP. Pendulum hardness (DIN 53157) and MEK rub test (ASTM D-5402) were performed to assess the curing rate of the coatings. Thermogravimetric analyses (TGA) of the UV-curing free films were conducted using a PerkinElmer Pyris 1 TGA model. The samples were heated from 30 to 700 °C at a heating rate of 10 °C/min under an air atmosphere. The current density–voltage characteristics of the cells (J-V) were measured using a Keithley 2400 source meter with a voltage scan rate of 10 mV/s. The light source used was AM1.5G filtered, and a light power of 100 mW/cm^2^ was employed for all J-V measurements. The light intensity was calibrated using a reference silicon solar cell with an area of 4 cm^2^ (Calab, Fraunhofer ISE, Germany). The encapsulated devices were tested in a solar climatic chamber (Atlas SEC 600) at room temperature and 60% relative humidity (RH), under a light intensity of 100 mW/cm^2^, for 48 h. OTR and WVTR were determined in units of grams per square meter per second (g/m^2^s). The film thickness applied was 100 μm. OTR was measured using the MOCON Ox-TRAN 2/21 oxygen permeability analyzer (ASTM D3985-05). WVTR was measured using the PERMETRAN W3/34 water vapor permeation analyzer (ASTM F1249-06).^32^

### 2.3 General Synthesis Procedure for AdAc- and ATES-Modified Epoxy Resin

To synthesize AdAc- and ATES-modified epoxy resin, a series of nine epoxy oligomer samples, namely, AdAc-MO1, AdAc-MO2, AdAc-MO3, AdAc-MO4, ATES-MO1, ATES-MO2, ATES-MO3, ATES-MO4, and ATES-MO5, were prepared in a two-step process with varying weight ratios of AdAc and ATES (Tables 1–2).

**Table 1:** Compositions^a^ of AdAc-MO Oligomer

sample	ER (mol)	AdAc (mol)	AcAc (mol)
AdAc-MO1	0.257	0.006	0.267
AdAc-MO2	0.254	0.009	0.259
AdAc-MO3	0.254	0.012	0.253
AdAc-MO4	0.254	0.015	0.246

a TEA and DMBA used 0.007 mol for all samples.

**Table 2:** Composition^a^ of ATES-MO Oligomer

sample	ER (mol)	ATES (mol)	AcAc (mol)
ATES-MO1	0.257	0.004	0.267
ATES-MO2	0.254	0.006	0.259
ATES-MO3	0.240	0.008	0.253
ATES-MO4	0.254	0.009	0.246
ATES-MO5	0.254	0.022	0.246

a TEA and DMBA used 0.007 mol for all samples.

Unlike AdAc-MO, a high ratio of modified material (ATES), such as 4.8%, has also been tried to improve adhesion.^33^ However, when the cross-link ratio increased excessively, the coating became brittle, and glass-to-glass adhesion decreased. The synthesis reactions were conducted in a 500 mL three-neck round-bottom flask equipped with a nitrogen inlet and a dropping funnel. In the first step, bisphenol A/F epoxy resin, along with either AdAc or ATES and TEA, was added to the flask. The reaction temperature was set at 90 °C, and the reaction progress was monitored. During the reaction, the acid value was observed to decrease to a range of 0.1–0.3 mg KOH/g within a 3 h period, indicating the completion of the reaction. According to Duan, when the conversion is calculated, the conversion occurs in the range of 98–99%.^34^ This step involves the opening of the epoxy resin’s oxirane ring and the subsequent connection of the polymer chains with either AdAc or ATES (Figures 1 and 2).

**Figure 1:**
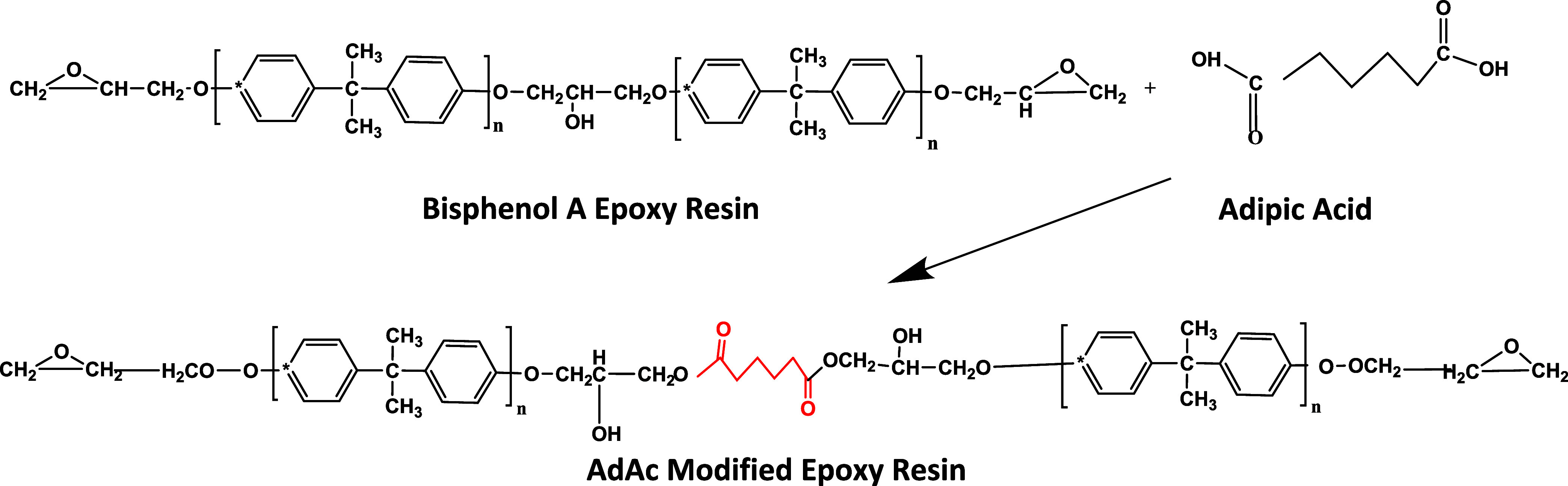
Reaction scheme of the AdAc-modified epoxy resin.

**Figure 2:**
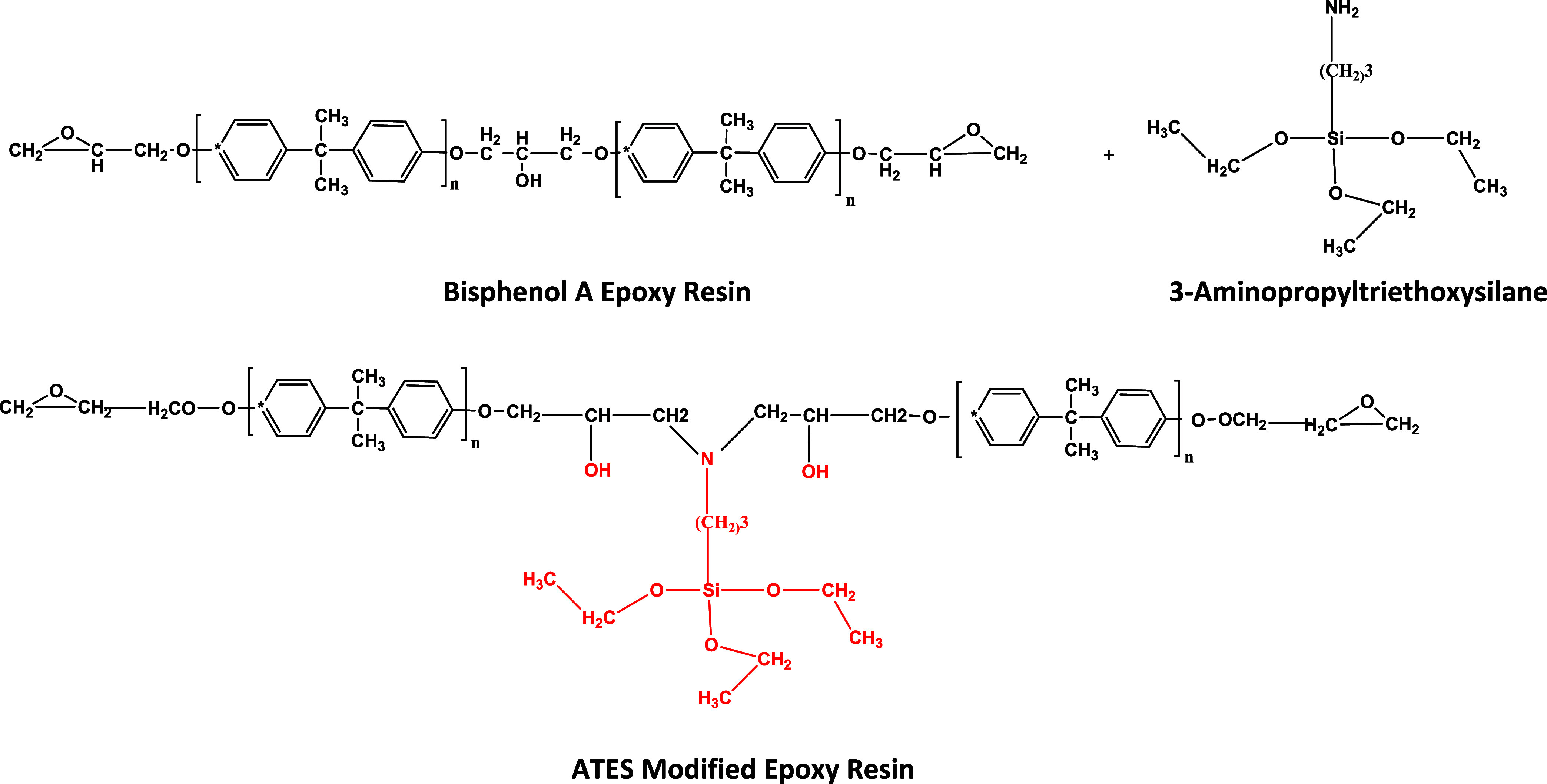
Reaction scheme of the ATES-modified epoxy resin.

During the second step, the reaction medium was cooled down from 90 to 60 °C. AcAc and DMBA were added dropwise to the well-stirred reaction mixture for a duration of 1 h, following the weight ratios indicated in Table 1. Once the addition of AcAc was complete, the reaction mixture was heated to 80 °C and maintained at this temperature for 14 h until the acid value of the resin decreased below 10 mg KOH/g. The total reaction time for this step was 21 h. Subsequently, the resulting epoxy acrylate oligomers modified with adipic acid (AdAc-MO) and ATES-silane (ATES-MO) were diluted with DPGDA at a weight ratio of 5:1. The reaction scheme outlining these procedures is depicted in Figures 3 and 4.

**Figure 3:**
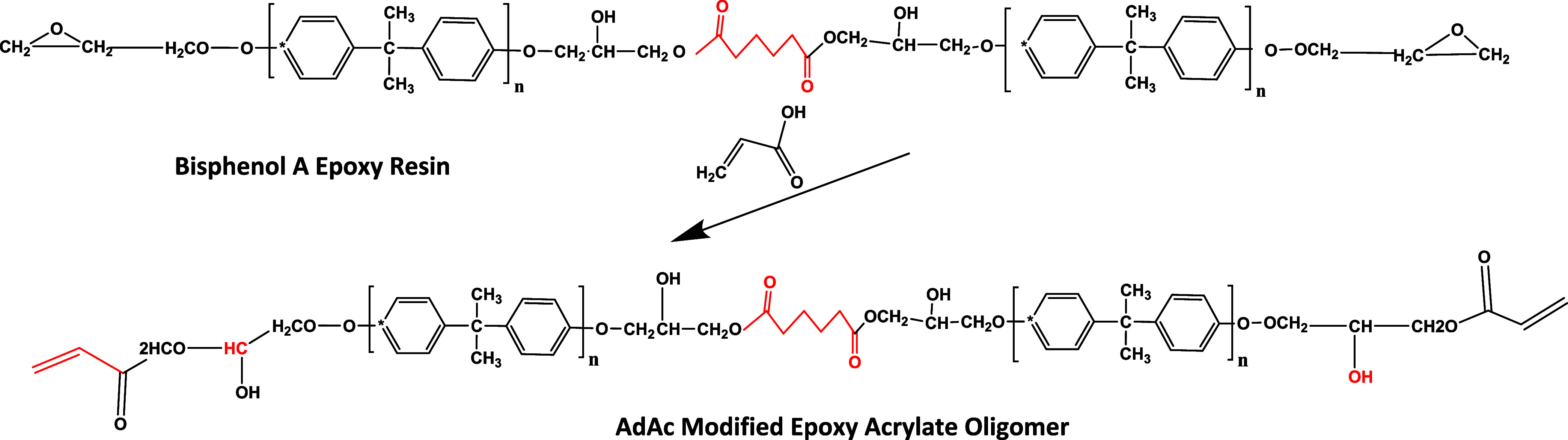
Reaction scheme of the AdAc-modified epoxy acrylate oligomer.

**Figure 4:**
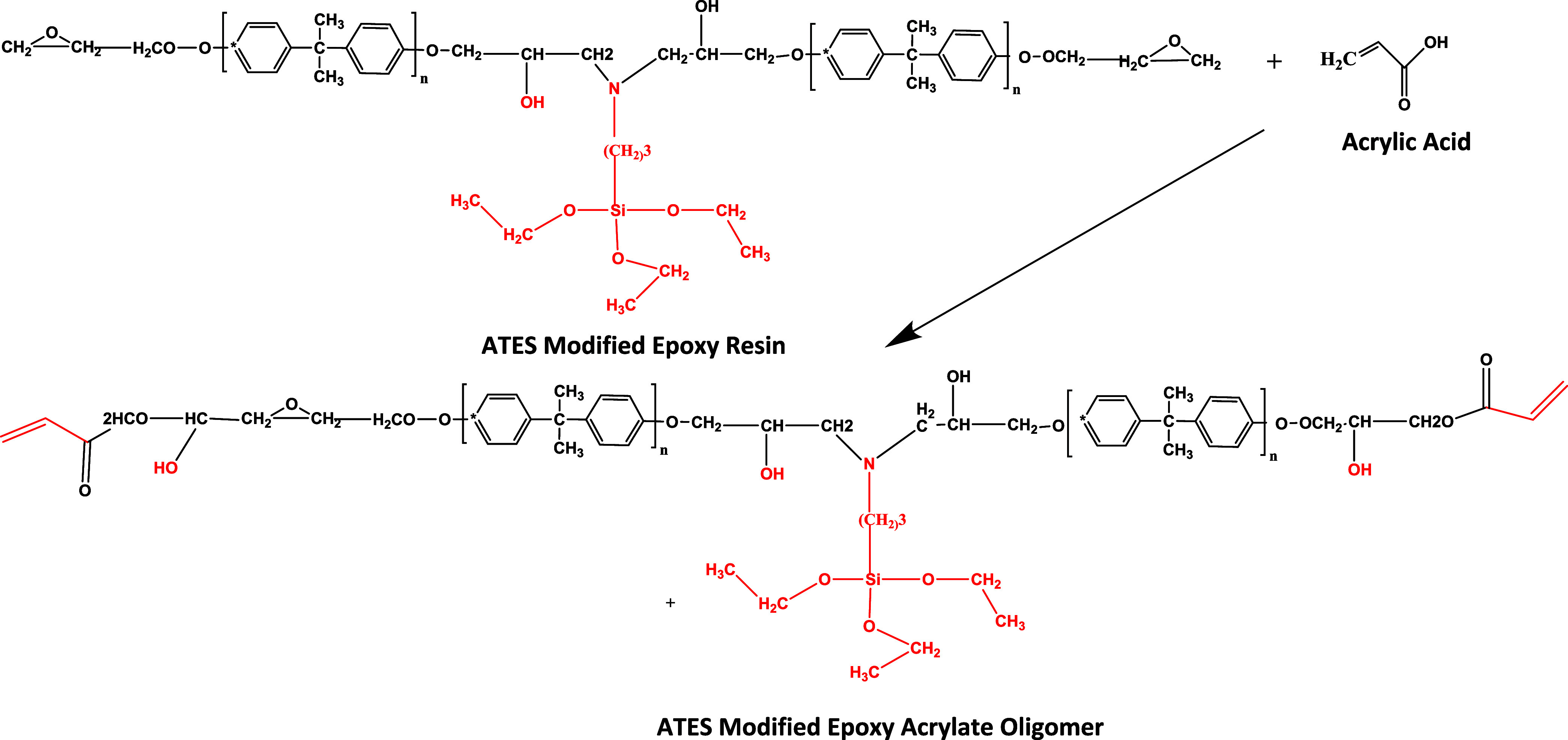
Reaction scheme of the ATES-modified epoxy acrylate oligomer.

### 2.4 Preparation of Coatings

The UV-curable formulations were prepared by adding AdAc-MO or ATES-MO (76.27 wt %) with DPGDA (19.29 wt %), photoinitiator (3.8 wt %), and defoamer (0.5%). Prior to the coating process, the aluminum and glass substrates were thoroughly cleaned using acetone to ensure surface cleanliness. Subsequently, the prepared formulations were applied onto the substrates by using an applicator. The applied coatings were then cured using a UV processor equipped with a medium-pressure mercury lamp, while maintaining a belt speed of 4m/min. The resulting film thickness on the substrates was approximately 30 μm (ASTM D 823).

### 2.5 Device Fabrication

The fabrication steps for the devices, along with all coating parameters, have been thoroughly described in our previous work.^35^ The active area of the devices was 0.095 cm^2^.

## 3 Results and Discussion

The general oligomer synthesis involves two reaction steps that were monitored through acid value determination and FTIR analyses. In the first step, bisphenol A epoxy resin was reacted with either AdAc or silane in the presence of a TEA catalyst to produce AdAc-modified epoxy resin (AdAc-ER) or ATES-modified epoxy resin (ATES-ER), respectively. During this reaction, the oxirane ring of the epoxy resin was opened, and AdAc or ATES moieties were incorporated into the epoxy resins. The acid value, initially around 16–18, decreased to 0.1–0.3 after a 3 h reaction period (Figure 5a,b). These results indicated the complete consumption of AdAc and ATES, confirming their reaction with epoxy resin. Additionally, the FTIR spectra (Figure 5c,d) provided further evidence of the reaction. The disappearance of the O–H (3000 cm^–1^) and C=O (1700 cm^–1^) peaks corresponding to AdAc after 3 h indicated their involvement in the reaction. Moreover, a new peak at 1725 cm^–1^ emerged, corresponding to the C=O ester carboxyl group present in the AdAc-ER (Figure 5c). In Figure 5d, the decrease in the epoxy ring peak after 3 h demonstrated a reduction in the number of epoxy rings in ATES-ER. In the second step, both AdAc-ER and ATES-ER were subjected to a reaction with AcAc at various ratios in the presence of TEA and DMBA catalysts to produce AdAc-MOs and ATES-MOs, respectively. This reaction involved the esterification of carboxyl and epoxide groups. Initially, the acid value was determined to be 145–150 (mg KOH/g), and the reactions were considered complete when the acid value decreased below 10 (Figure 6a,b). Conversion was calculated according to Duan,^34^ and a conversion rate of 93–94% was achieved. The successful progress of the reactions was confirmed by the presence of characteristic peaks in the FTIR spectra, including an OH peak at approximately 3450 cm^–1^, an acrylate peak at 1634 cm^–1^, and C=O ester peaks at 1725 cm^–1^ (Figure 6c,d).^36^

**Figure 5:**
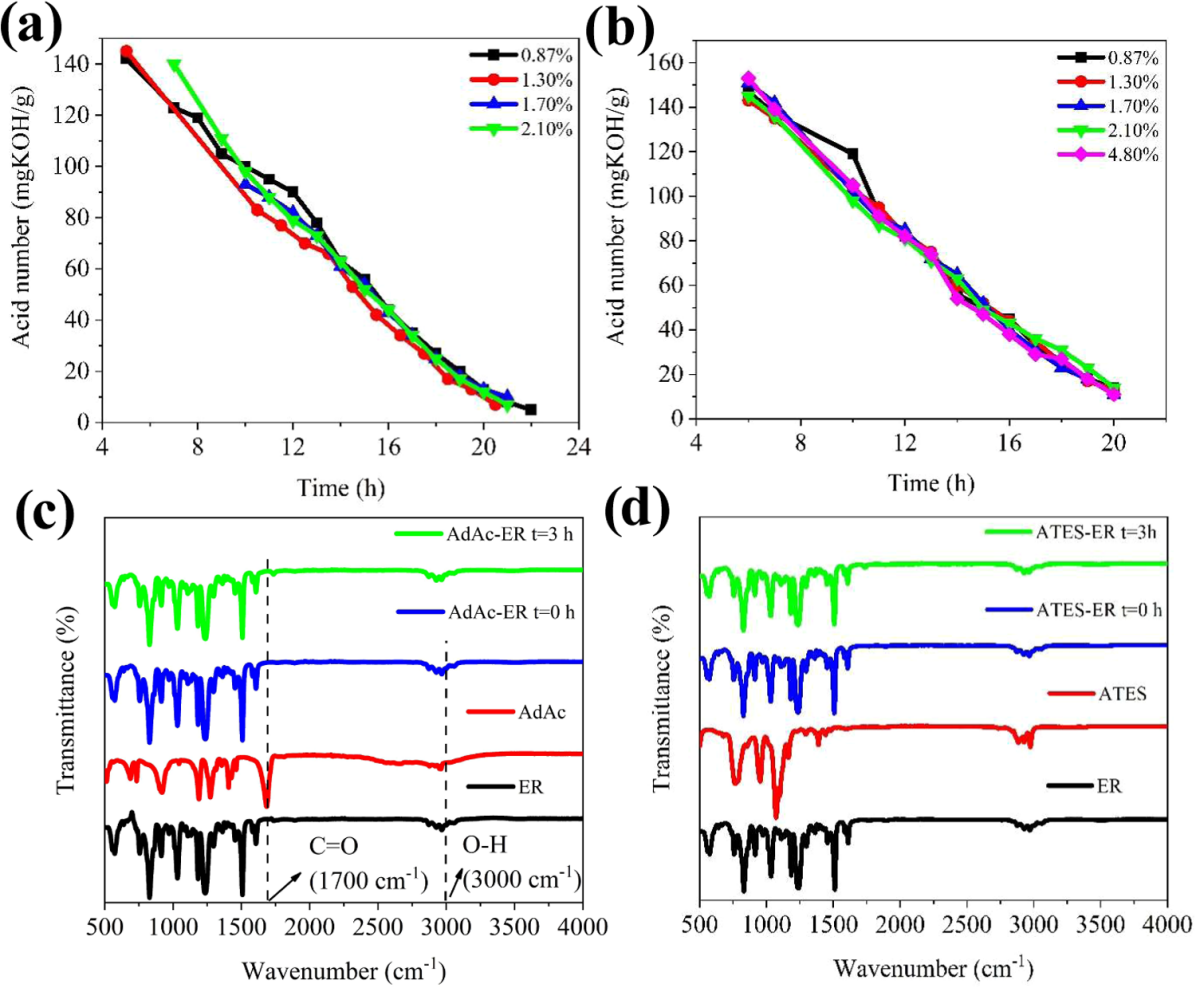
Acid value graph versus reaction time modified epoxy resin: (a) AdAc-ER and (b) ATES-ER epoxy resin. FTIR spectra (c) AdAc-ER and (d) ATES-ER resins in *t* = 0 and 3 h reaction time.

**Figure 6:**
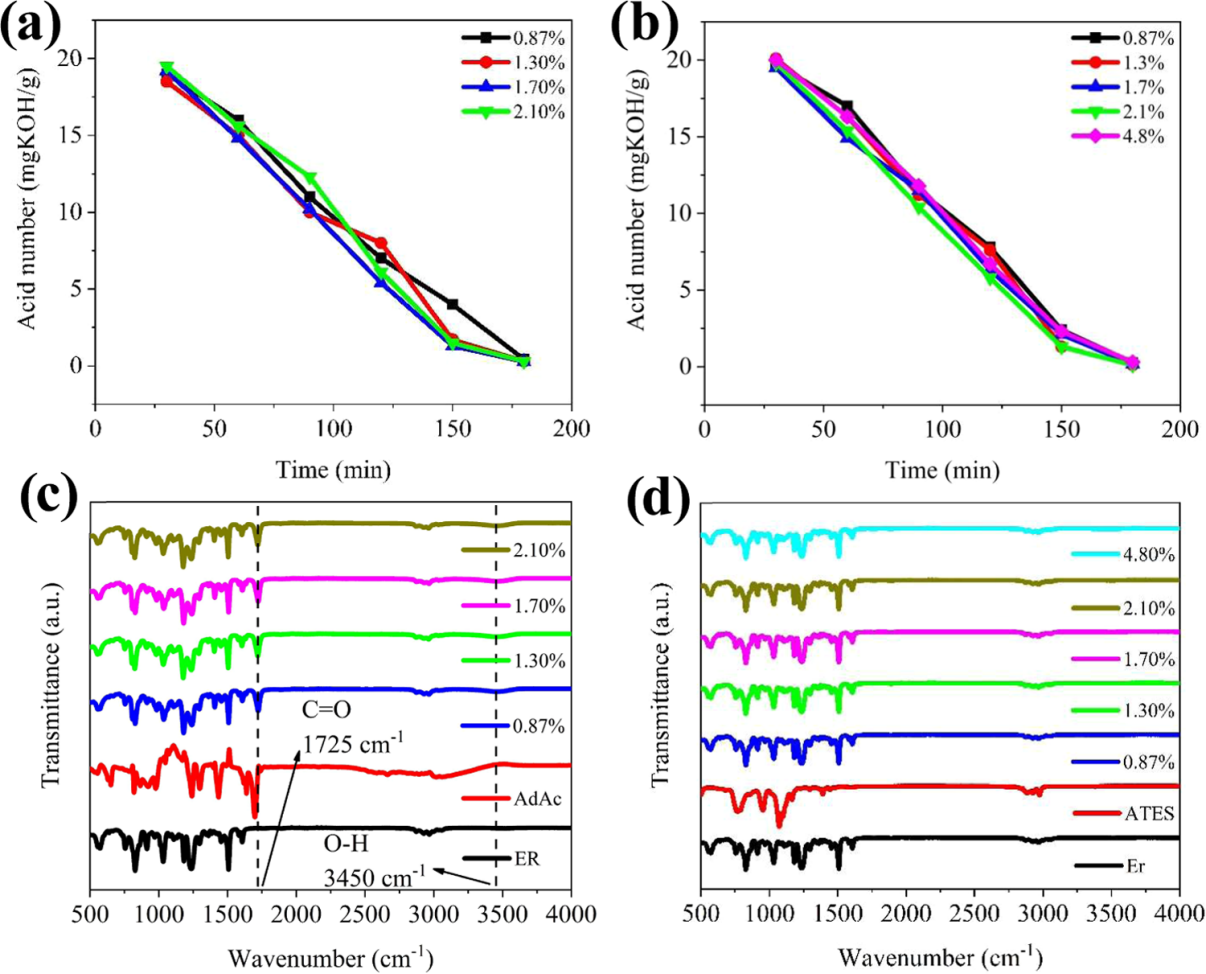
Acid value graph versus reaction time for modified epoxy resins: (a) AdAc-MOs and (b) ATES-MOs. FTIR spectra (c) AdAc-MOs; (d) ATES-MOs in different ratios of AcAc/ER (w/w).

In the ^1^H NMR spectra (in CDCl_3_) of both AdAc-MO and ATES-MO, new peaks were observed at 5.9–6.5 ppm, which corresponded to the protons of the acrylate groups (Figures S1 and S2). Additionally, peaks at 1.65 and 6.7–7.2 ppm were identified as the aliphatic (−CH_3_) and aromatic (phenyl) protons, respectively. Moreover, new peaks appeared at 4.28 ppm, indicating the presence of protons on the carbon adjacent to the hydroxyl group (−CH–OH). In the ^1^H NMR spectra of AdAc-MO, a singlet peak at 2.4 ppm represented the hydroxyl proton, while the alkyl group proton of acrylates was observed at 1.25 ppm. However, the hydroxyl proton peak was absent in the ^1^H NMR spectra of ATES-MO. These findings corroborate the results obtained from FTIR analysis, confirming the progress of the reactions. Following the synthesis of the new oligomers, coating processes were conducted to evaluate the impact of modification on the physical and mechanical properties of the cross-linked polymers. The physical and mechanical properties of the UV coatings prepared using the synthesized AdAc-MOs and ATES-MOs are presented in Tables 3 and 4, respectively. Analysis of the viscosity values of the oligomers revealed that at low concentrations of AdAc and ATES, the viscosities increased. However, at higher concentrations, the viscosities remained constant or even decreased in some samples (e.g., AdAc-MO4). Notably, the oligomers AdAc-MO and ATES-MO1 exhibited viscosities lower than those of the standard (unmodified) oligomer. Furthermore, the hardness of the coatings was assessed by using the pendulum hardness test method. Table 3 shows that, except for AdAc-MO4 with the highest modification rate, the hardness of the AdAc-modified cross-linked polymers was lower than that of the standard. Conversely, the ATES-modified cross-linked polymers displayed higher hardness values compared to the standard (Table 4).

**Table 3:**
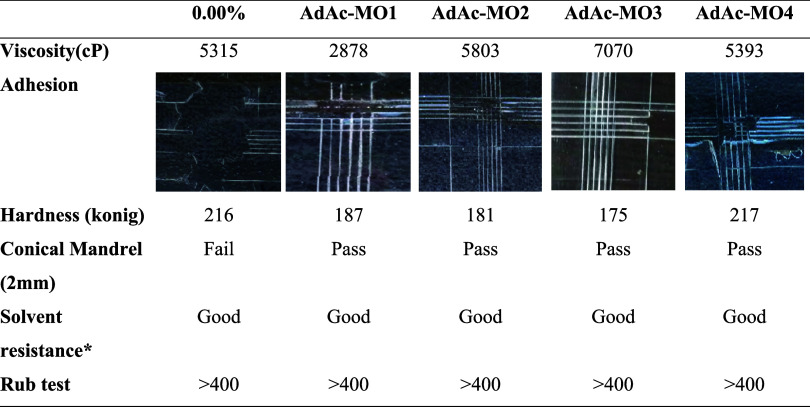
UV Coatings Performance Test Result (AdAc-MOs)

* 2 min. Acetone.

**Table 4:**
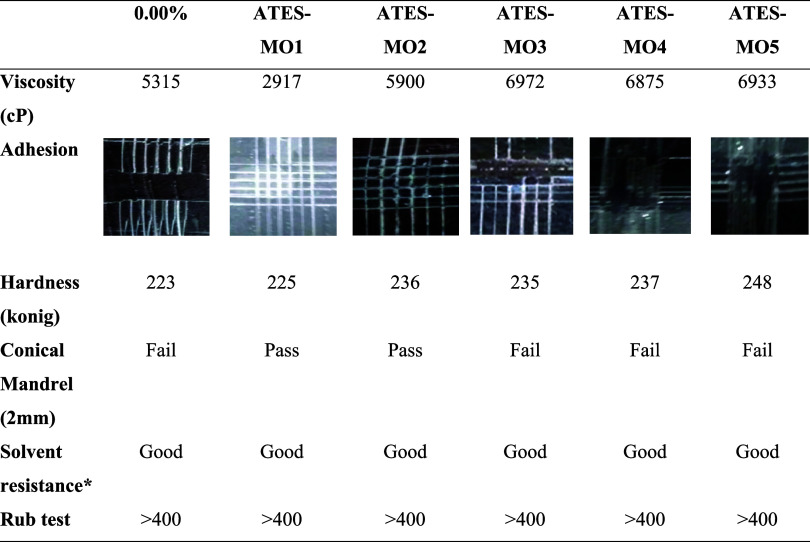
UV Coatings Performance Test Result for ATES-MO

* 2 min. Acetone.

The solvent resistance of all polymer coatings was evaluated through methyl ethyl ketone (MEK) rubbing and acetone tests. After the coatings were subjected to 400 double rubs in the MEK rubbing test, no deformation was observed on the surface of any modified polymers, similar to the standard polymer. Additionally, in the acetone test, all coatings were exposed to the solvent for 2 min, and at the end of the test, the surfaces of all coatings remained undamaged. These results confirm that the modified polymer coatings exhibit chemical resistance comparable to that of the standard cross-linked polymer, supporting their suitability for encapsulation applications. The adhesion of the coating materials was evaluated by using the cross-cut adhesion test method. It was observed that the adhesion increased with higher AdAc ratios (Table 3). However, unmodified acrylate and AdAc-MO1 and AdAc-MO2 did not adhere sufficiently to the glass surface. Improved adhesion was achieved by increasing the AdAc ratio, which led to lower cross-linked density and reduced shrinkage. Notably, the unmodified coating could be completely removed from the surface. The results of the adhesion test were further supported by a conical mandrel analysis. This test assesses the resistance of a dry film to cracking or detachment from a flexible substrate when it is bent around a cylindrical mandrel. As a reference test photo, Figure S3 shows photos of samples that passed and failed the test. The AdAc-MO3 (0.087%) sample passed the test successfully, while other AdAc-modified samples passed too, but the samples have a little scratch. Therefore, it was demonstrated that AdAc-MO3 exhibited better flexibility compared to the others. Regarding the ATES-modified polymer, increasing the modification ratio resulted in higher hardness due to increased cross-linking. However, the adhesion was poor due to film hardening issues. Thus, the coating with the lowest cross-linking exhibited the best flexibility.^36,37^ The conical mandrel test results confirmed the findings of the adhesion test, as the dry film was completely removed from the surface as the modification rate increased. The hardness of a coating primarily depends on the chain flexibility of the molecules and cross-linking density.^20,38^ While hard coatings offer better scratch resistance, their rigidity can lead to cracking, which is a disadvantage.^39,40^ Coating gloss is a complex phenomenon influenced by the interaction between light and the surface of the coating.^41^ These properties are closely related to the cross-linking density in the polymeric film and the chemical structure of the formulation.

Thermal analyses were conducted to evaluate the phase transition properties of all polymers. *T*_g_ of the encapsulation material primarily depends on the flexibility of the polymer structure.^39^ The unmodified epoxy resin possesses an amorphous structure with *T*_g_ of approximately 57 °C. As the AdAc modification ratio increases, the *T*_g_ values of the cross-linked polymers decrease. However, a similar trend is not observed for the ATES modification. Instead, as the ATES modification ratio increases, the *T*_g_ values of the corresponding polymers increase (Figure 7a–c). This indicates that the siloxane groups present in ATES contribute to the cross-linking process. Since ATES-modified oligomers form more cross-links during curing compared to AdAc-modified oligomers, the rigidity of the polymer and intermolecular interactions increase, resulting in higher *T*_g_ values. The thermal degradation behavior of the encapsulation coatings was examined by using TGA (Figure 7b–d). It was observed that the modification of the encapsulant coatings did not significantly affect their thermal stability. The initial decomposition occurred between 150 and 170 °C and was attributed to the reactive diluent, photoinitiator, moisture, and residual solvent in the film. In the derivative of the TGA curves, two characteristic weight loss peaks were observed (Temperatures 1 and 2). The first peak at 300–360 °C seems to be the main decomposition corresponding to the cleavage of polymer chains. The second decomposition, which occurs at 450–490 °C, may be attributed to the further fragmentation of cleaved polymer chains. Since the degradation temperature exceeds 150 °C, which is well above the maximum operating temperature of a solar cell under sunlight (∼100 °C), this does not pose a limitation for the use of our epoxy-based encapsulation material.^42^ All modified and unmodified coating samples exhibited a 10% mass loss between 280 and 320 °C. Additionally, the char yield increased with both AdAc and ATES ratios, with ATES-MOs showing higher char yields compared to AdAc-MOs (Table 5).

**Figure 7:**
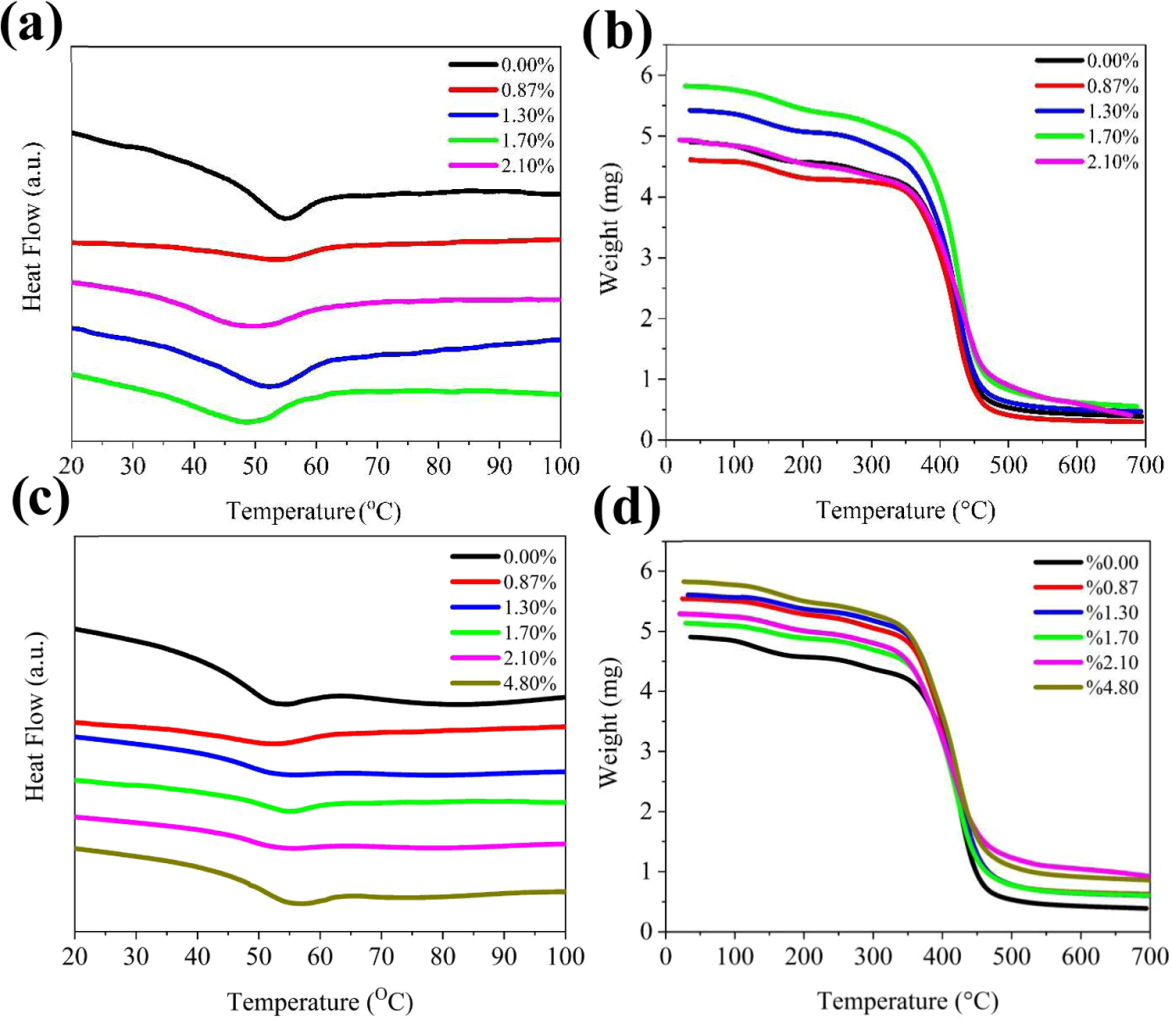
AdAc-MO (a) DSC and (b) TGA diagram; ATES-MO (c) DSC; and (d) TGA diagram.

**Table 5:** Thermal Properties of AdAc-MOs

sample	*T*_g_ (°C)	%10 mass loss (°C)		temperature 1 (°C)		temperature 2 (°C)	char yield (%)
0.00%	54.70	321.00		343.00		476.00	7.52
AdAc-MO1	53.70	341.00		355.00		483.00	6.50
AdAc-MO2	52.40	294.00		356.00		475.00	8.71
AdAc-MO3	48.20	290.00	349.00		469.00		9.50
AdAc-MO4	49.10	271.00	341.00		483.00		8.26
ATES-MO1	54.00	322.00	331.00		453.00		11.25
ATES-MO2	53.00	321.00	333.00		455.00		11.29
ATES-MO3	55.00	322.00	335.00		458.00		11.89
ATES-MO4	55.30	311.00	332.00		455.00		14.79
ATES-MO5	56.00	314.00	327.00		450.00		17.60

After mechanical and physical characterization of the modified oligomers and corresponding cross-linked polymers was conducted, they were utilized as encapsulation materials in solar cell applications to assess their impact on device performance and lifetime stability. The n-i-p devices were structured as follows: FTO/Li-treated c-TiO_2_/triple-cation perovskite/Spiro-OMeTAD/Au. The encapsulation materials were applied and cured by using a UV curing system inside a glovebox on the triple-cation PSCs. Stability tests were conducted for 48 h in a solar climatic chamber under 100 mW/cm^2^ irradiation at 25 °C and 60% RH. A standard cell without encapsulation was also used as a control cell. J-V graphs and electrical parameters were obtained under a reverse bias. Prior to encapsulation, the PCEs of all solar cells were determined inside the glovebox. Subsequently, the cells were encapsulated, and the PCEs were recorded again to evaluate the immediate effect of encapsulation. Finally, at the end of the aging test, all cells were tested to assess the performance of the encapsulation. The results are presented in Table 6. The efficiency reductions of the devices coated with AdAc-MOs are shown in Figure 9a. The lowest efficiency was observed in the unmodified epoxy acrylate AdAc-MO0-based polymer-coated devices, while the highest efficiency was achieved in the AdAc-MO3-based polymer-coated ones. The OTR and WVTR values were measured to evaluate the encapsulation performance of the modified and unmodified polymers.^19^ These parameters indicate the ability of the encapsulated PSCs to maintain device stability by protecting against moisture and oxygen degradation. The moisture and oxygen permeabilities of AdAc-MOs were determined at 23 and 38 °C, respectively. The WVTR and the OTR values of the AdAc-MO-based epoxy encapsulant materials were found to be lower than those of unmodified epoxy acrylate, confirming an improvement in barrier properties. In the literature, epoxy acrylate WVTR values have been reported as 16 g/m^2^day under comparable conditions.^19^ In our study, the best-performing encapsulant, AdAc-MO3, exhibited significantly lower OTR and WVTR values of 5.10 cm^3^/m^2^day and 7.46 gr/m^2^day, respectively (Table S1). These results confirm that the modification enhanced the encapsulation efficiency by reducing oxygen and water vapor diffusion. The observed improvements in barrier properties correlate with efficiency measurements, as shown in Figures S4 and S5. A schematic representation of the encapsulation methods of the produced perovskite solar cells is presented in Figure 8. The solar cell encapsulated with AdAc-MO3 exhibited the lowest current degradation and the highest fill factor (FF) after the aging test (Table 6). Furthermore, commercial organic electronic devices typically require WVTR values below 10^–3^ g/m^2^day for long-term stability. While perovskite solar cells do not yet have an established WVTR/OTR standard, our results demonstrate a significant improvement over that of unmodified epoxy acrylate and align with performance expectations for enhanced encapsulation materials.

**Table 6:** Best I–V Characteristic AdAc-MO and ATES-MO-Based Encapsulant Material

	before encapsulation PCE (%)	after encapsulation PCE (%)	after stability test PCE (%)
control	19.0	5.70	3.9
AdAc-MO 1.70%	17.9	15.0	4.8
ATES-MO 0.87%	14.3	13.7	10.6

**Figure 8:**
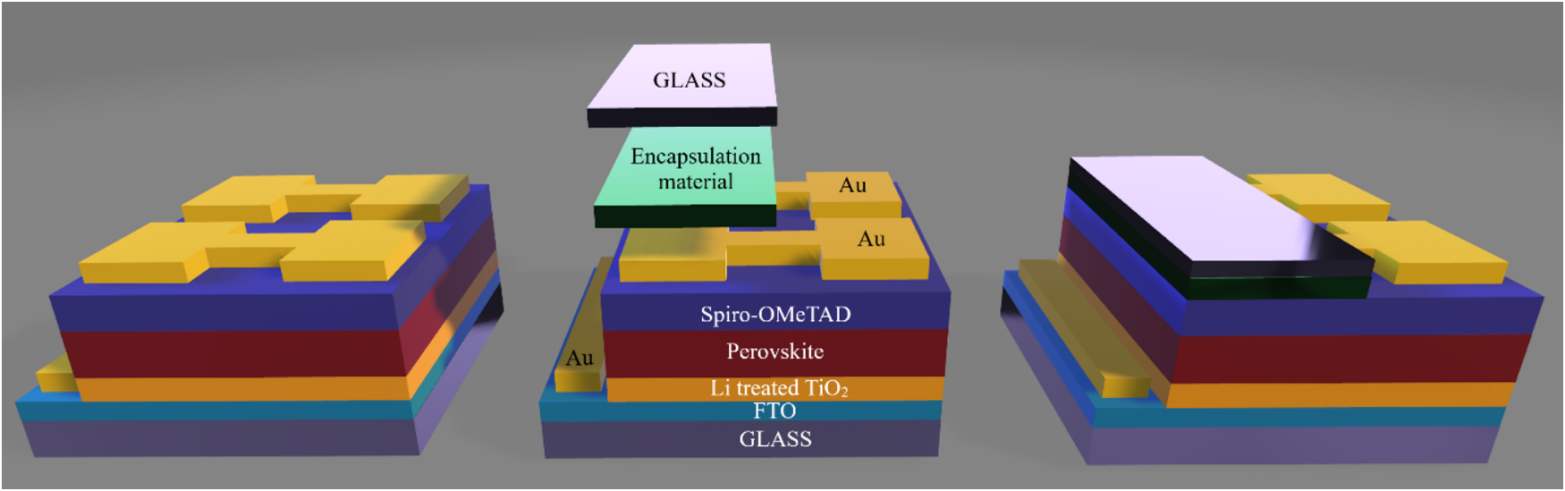
Schematic illustration of an encapsulated PSC.

The efficiency reductions of solar cells encapsulated with ATES-MOs are presented in Figure 9c. The lowest efficiency drop was observed in the cells encapsulated with ATES-MO4 and ATES-MO5, while the best results were achieved with ATES-MO3. Figures S4–S5 and Tables S3–S4 show that the J-V characteristics were consistent with the efficiency results. The moisture and oxygen permeabilities of the ATES-MO-based polymer encapsulants were determined at 23 and 38 °C, respectively. The WVTR and OTR values of the ATES-MO-based polymer encapsulant were found to be lower than those of the unmodified epoxy acrylate. However, as shown in Figure 9d, after modification, all encapsulant films became very rigid due to the increased cross-link density and were prone to breakage when detached from the glass surface. Despite the brittleness of the films, low WVTR values were obtained. The OTR and WVTR results align with the efficiency measurements (Table S2). However, the desired stability of the devices could not be achieved due to the increased stiffness caused by the addition of silane, which increased the cross-linking and branching rate in the oligomer structure. To mitigate the detrimental effects of UV light on the encapsulation material and enhance solar cell performance, epoxy oligomers were cured using a visible light (VL) photoinitiator (ISOVIII).^33,43^ The UV–vis absorption spectrum of this photoinitiator demonstrates its efficiency in initiating the polymerization process under visible light exposure (Table 7).^44^

**Figure 9:**
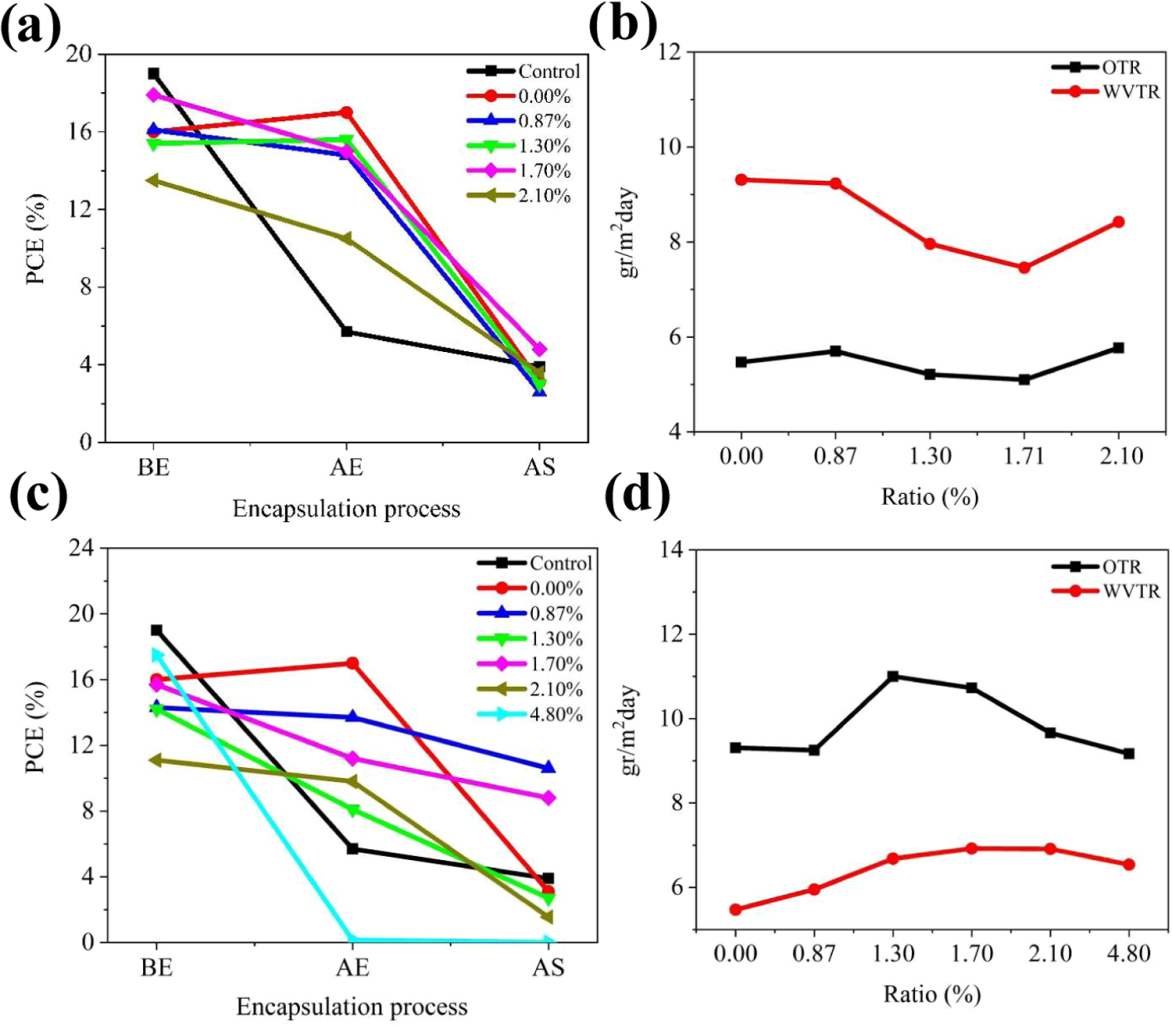
(a) Efficiency performance of AdAc-MOs encapsulation. (b) OTR and WVTR measurement of AdAc-MO-based polymers. (c) Efficiency performance of ATES-MOs encapsulation. (d) OTR and WVTR measurement of ATES-MO-based polymers (BE: before encapsulation, AE: after encapsulation, AS: after stability test).

**Table 7:** Device Performance under UV and VL Encapsulants

encapsulant	light source	PCE before encapsulation (%)	PCE after encapsulation (%)	PCE drop (%)
AdAc-MO3 based	VL	14.2	13.1	8.0
	UV	17.9	15.0	16.0
ATES-MO3 based	VL	17.1	13.4	21.6
	UV	15.7	11.2	28.7

## 4 Conclusions

In this study, we successfully synthesized and characterized modified epoxy acrylate oligomers incorporating AdAc and ATES to enhance the mechanical, thermal, and barrier properties of encapsulation materials for organic electronics, particularly PSCs. The results demonstrated that AdAc-MO3, with an optimal balance between hardness, flexibility, and barrier performance, significantly outperformed unmodified epoxy acrylate and other modified oligomers. Notably, AdAc-MO3 exhibited an impressive reduction in WVTR and OTR, highlighting its potential for providing long-lasting protection for PSCs under environmental stress conditions. The enhanced thermal stability of ATES-MO3 was also observed, although it presented challenges in achieving the ideal balance of adhesion and flexibility required for optimal encapsulation. While ATES-MO3 demonstrated superior thermal properties, its higher cross-linking density led to reduced adhesion and flexibility, which makes it less suitable for certain encapsulation applications compared to AdAc-MO3. However, the highest device stability was achieved with 0.87% ATES-MO1 (77.3% of the initial PCE), accompanied by a reduction in the OTR and WVTR values. Therefore, future modification studies should focus on testing lower ratios of ATES. Additionally, the study revealed that the use of visible light curing, enabled by a synthesized photoinitiator (ISOVIII), provides a sustainable and effective alternative to UV curing, offering improved device stability and lower efficiency loss. These findings underscore the importance of modifying encapsulant materials to achieve enhanced stability and performance of PSCs, which are essential for their commercial viability. Overall, this research not only contributes to the development of high-performance encapsulation materials but also provides valuable insights into the optimization of material properties for advanced organic electronics.

## Supplementary Material



## Notes

^1^H NMR spectra of AdAc-MO and ATES-MO, J-V plots of the PSCs under reverse bias, photovoltaic parameters of PSCs based on AdAc-MO and ATES-MO, OTR and WVTR values of AdAc-MOs and ATES-MOs, and conical mandrel test result of 0.00% ATES-MO and ATES-MO 0.87% (PDF)
