# Supplementary material for: Improved Mechanical and Physical Properties of Epoxy Acrylate Oligomers by Chemical Modification for the Effective Encapsulation of the Triple-Cation Perovskite Solar Cells
Source: ACS Omega. 2025 May 7;10(19):19723–34. doi: 10.1021/acsomega.5c00860 (PMC12096231; doi:10.1021/acsomega.5c00860)
Supplement: Supplementary file 1 [file ao5c00860_si_001.pdf]

Supporting Information

Improved Mechanical and Physical Properties of Epoxy Acrylate Oligomers by Chemical Modification for Effective Encapsulation of The Triple-Cation Perovskite Solar Cells

*Bahar Tosun Ercan [a,b], Adem Mutlu [a], Sirin Siyahjani Gultekin [a], Burak Gultekin [a], Haluk Dincalp [c], Ceylan Zafer [a,]**

[a] Ege University, Solar Energy Institute, 35100, Izmir, Türkiye

[b] Kubilay Paint Industry, 35800, Izmir, Türkiye

[c] Manisa Celal Bayar University, Department of Chemistry, Faculty of Arts and Science, Yunus Emre, 45140, Manisa, Türkiye

**Corresponding authors: ceylan.zafer@ege.edu.tr*

This file includes:

Supporting Figure S1-S5

Supporting Table S1-S4

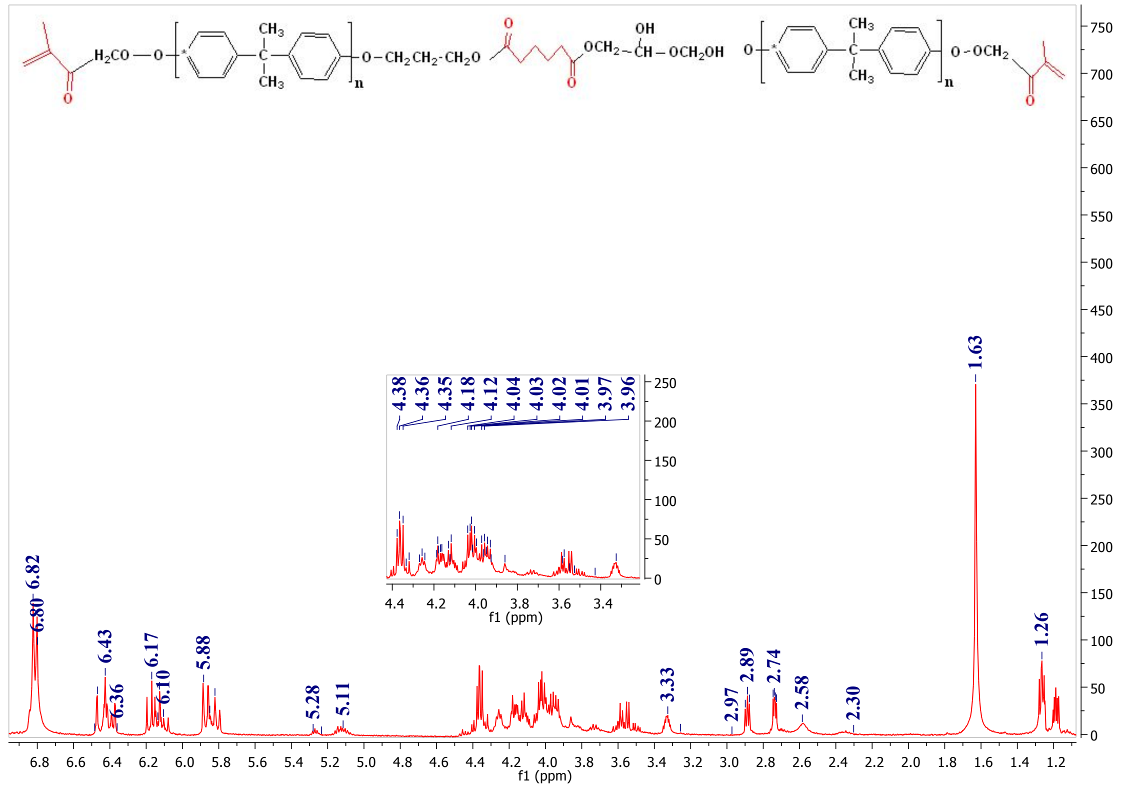

Figure S1. ^{1}H-NMR spectra of AdAc-MO.

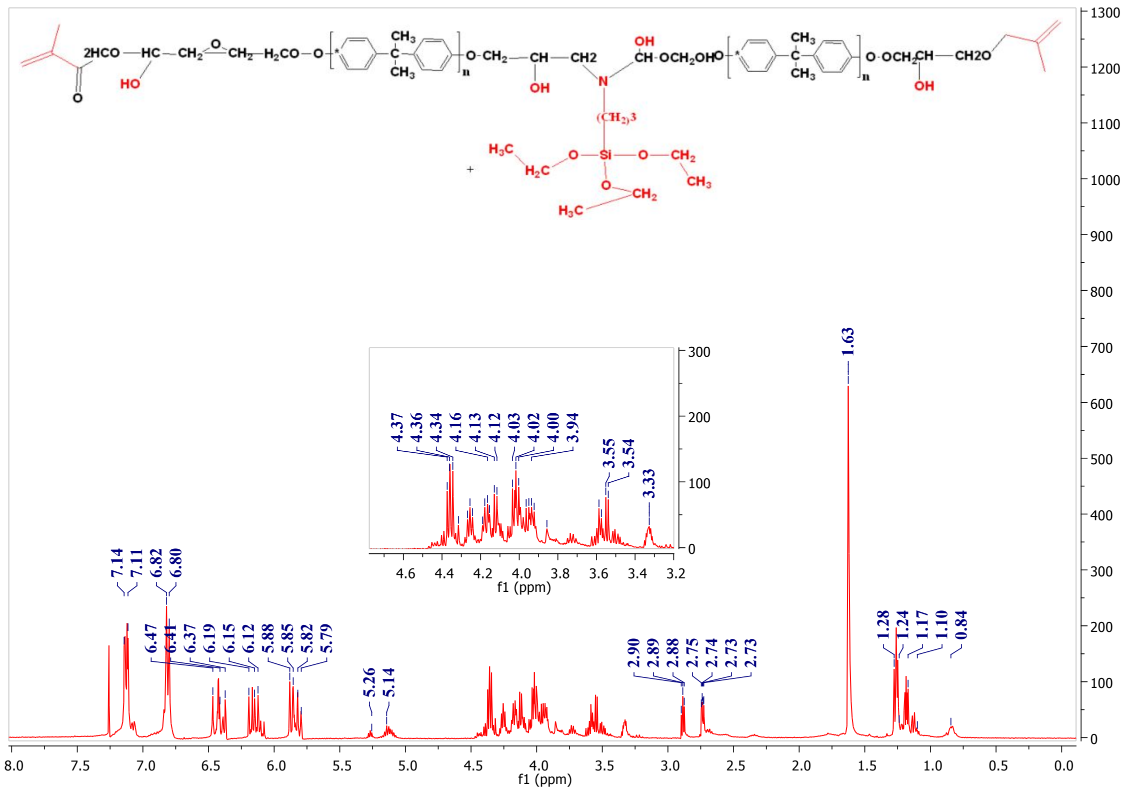

Figure S2. ^{1}H-NMR spectra of ATES-MO.

FAIL	PASS
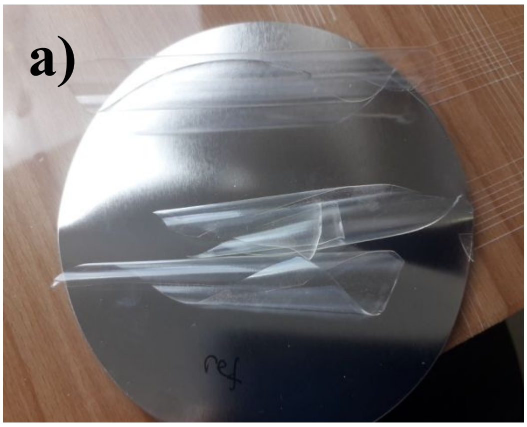	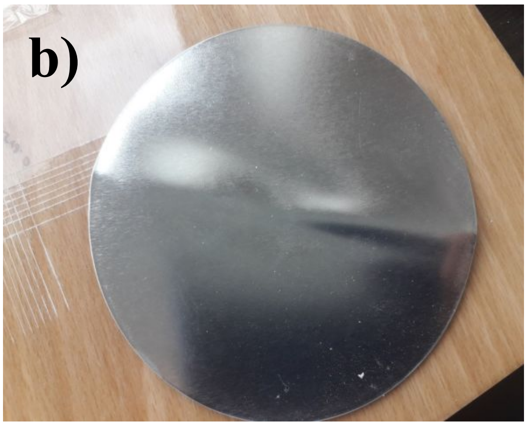

Figure S3. Conic Mandrel Test Result of a) 0.00% ATES-MO, b) ATES-MO 0.87% based materials.

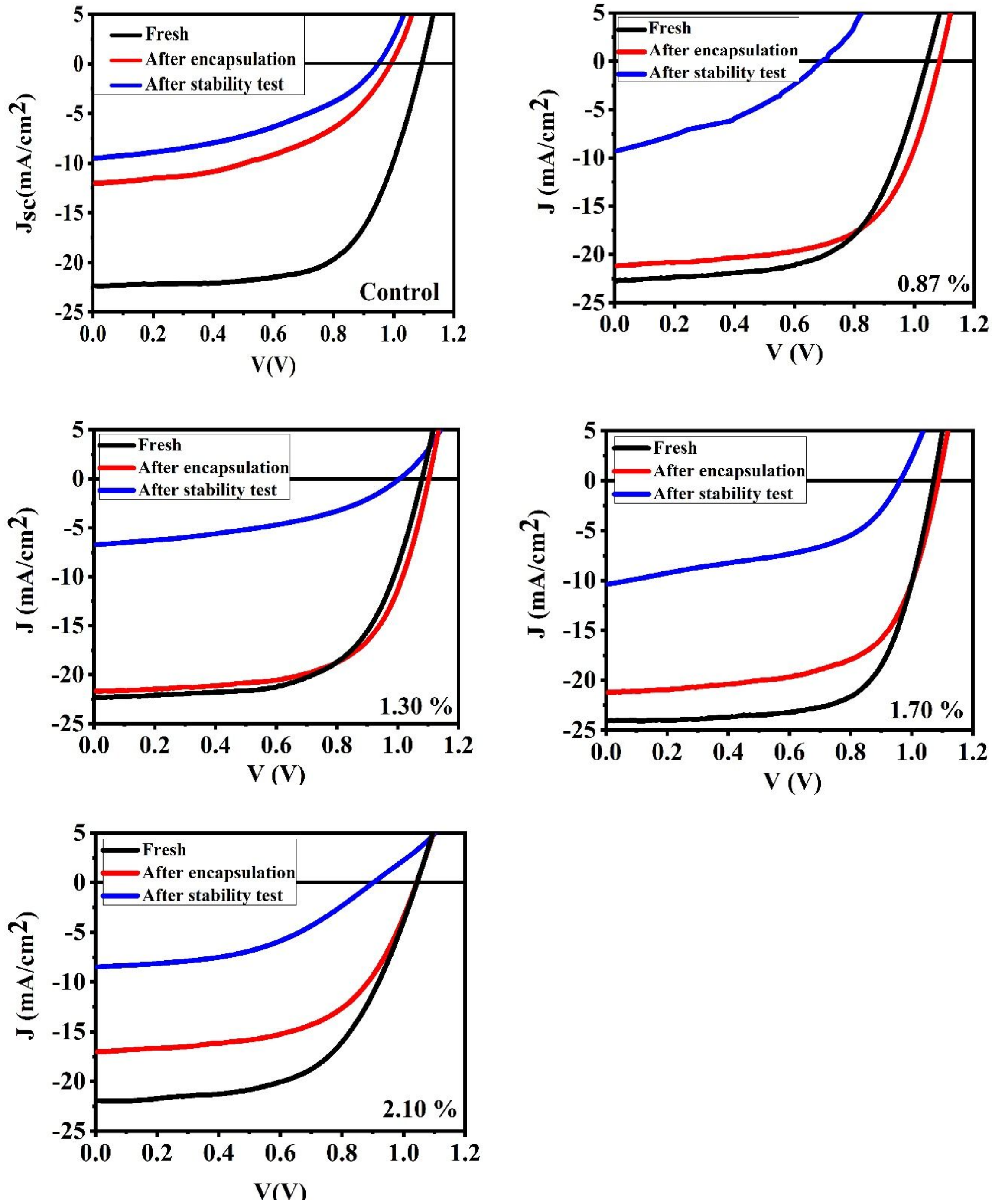

Figure S4. Current density-voltage (J-V) characteristics of PSCs encapsulated with AdAc-MOs before and after encapsulation and after stability test.

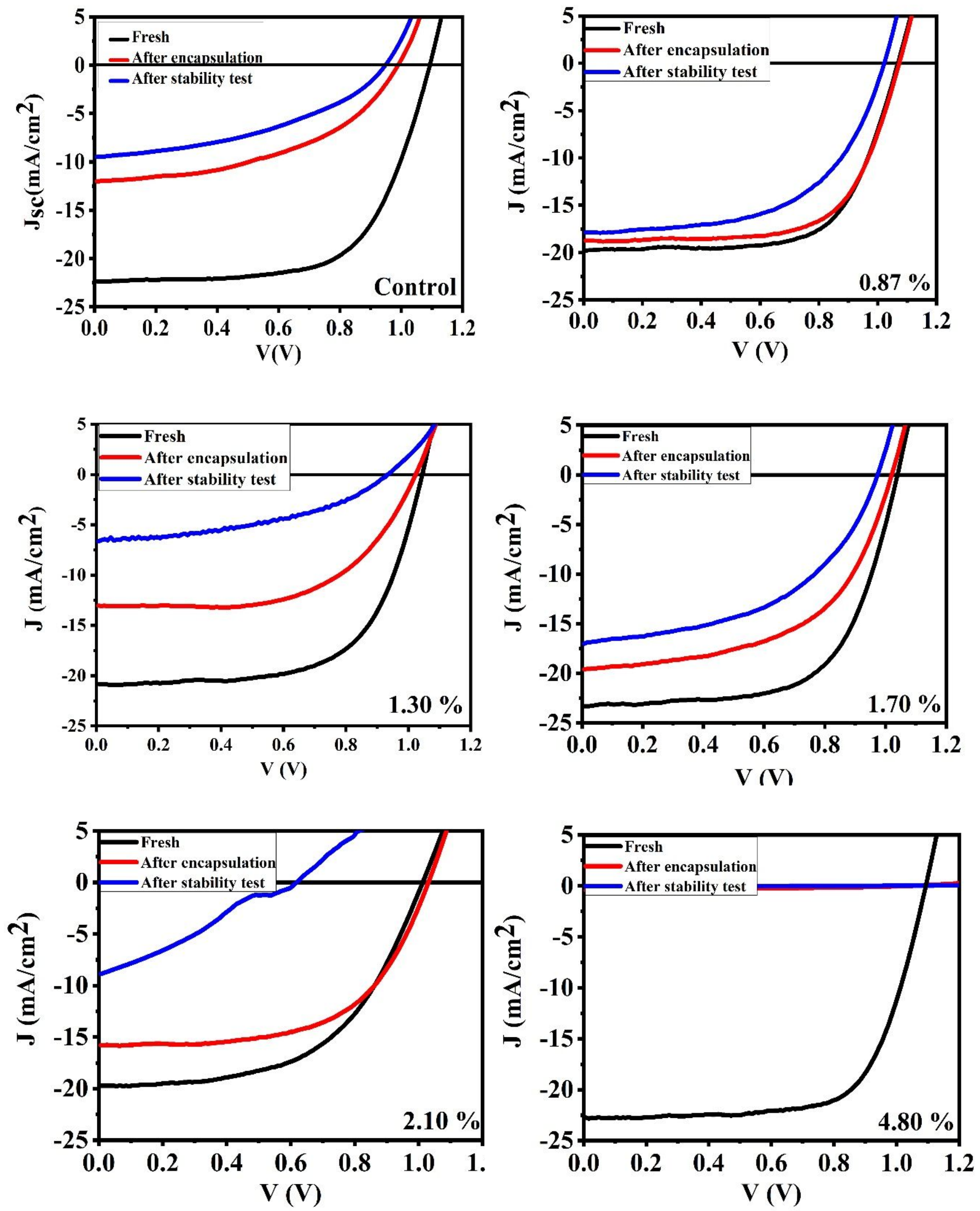

Figure S5. Current-voltage (J-V) characteristics of PSCs encapsulated with ATES-MOs before and after encapsulation and after stability test.

Table S1. OTR and WVTR values of AdAc-MOs based polymers.

Sample	OTR<br>g/m^2.day	WVTR<br>g/m^2.day
**%0.00**	9.31	5.47
**%0.87**	9.23	5.70
**%1.30**	7.96	5.21
**%1.71**	7.46	5.10
**%2.10**	8.42	5.77

Table S2. OTR and WVTR values of ATES-MOs based polymers.

Sample	OTR<br>g/m^2.day	WVTR<br>g/m^2.day
**%0.00**	9.31	5.47
**%0.87**	9.25	5.95
**%1.30**	11.00	6.68
**%1.71**	10.73	6.71
**%2.10**	9.17	6.54
**%4.80**	9.66	6.91

Table S3. Photovoltaic parameters of PSCs based on AdAc-MO encapsulant material.

Sample	Fresh				After Encapsulation				After Stability Test			
	J_{SC} (mA/ cm²)	V_{OC} (mV)	FF (%)	PCE (%)	J_{SC} (mA/ cm²)	V_{OC} (mV)	FF (%)	PCE (%)	J_{SC} (mA/ cm²)	V_{OC} (mV)	FF (%)	PCE (%)
**Control**	24.5	1110	69.9	19.0	12.0	990	48.0	5.70	9.6	950	42.8	3.9
**%0.00**	21.9	1072	57.9	16.0	14.0	1000	58.2	17.0	11.6	736	36.0	3.1
**%0.87**	23.0	1050	66.7	16.1	21.7	1085	63.0	14.8	9.3	685	41.0	2.6
**%1.30**	22.5	1085	63.1	15.4	22.0	1100	64.5	15.6	6.6	1010	45.0	3.0
**%1.70**	23.8	1080	69.6	17.9	21.4	1090	64.3	15.0	10.7	965	47.0	4.8
**%2.10**	21.6	1050	59.5	13.5	17.1	1045	58.8	10.5	8.7	905	46.0	3.6

Table S4. Photovoltaic parameters of PSCs based on ATES-MO encapsulant material.

Sample	Before Encapsulation (BE)				After Encapsulation (AE)				After Stability Test (AS)			
	J_{SC} (mA/ cm²)	V_{OC} (mV)	FF (%)	PCE (%)	J_{SC} (mA/ cm²)	V_{OC} (mV)	FF (%)	PCE (%)	J_{SC} (mA/ cm²)	V_{OC} (mV)	FF (%)	PCE (%)
**Control**	24.5	1110	69.9	19.0	12.0	990	48.0	5.70	9.6	950	42.8	3.9
**%0.00**	21.9	1072	57.9	16.0	14.0	1000	58.2	17.0	11.6	736	36.0	3.1
**%0.87**	19.7	1070	67.8	14.3	18.7	1070	68.5	13.7	17.8	1025	58.1	10.6
**%1.3**	20.8	1045	65.3	14.2	13.1	1020	60.6	8.1	6.7	935	43.1	2.7
**%1.7**	23.3	1045	64.5	15.7	19.6	1020	56.0	11.2	17.0	975	53.1	8.8
**%2.1**	19.8	1010	55.5	11.1	15.8	1030	60.2	9.8	9.0	620	27.8	1.55
**%4.8**	22.8	1095	70.1	17.5	0.40	1030	36.4	0.15	0.04	785	22.3	0.007

(BE: before encapsulation, AE: after encapsulation, AS: after stability test.)
